# A Novel High-Power Dual-Band Coupled-Line Gysel Power Divider with Impedance-Transforming Functions

**DOI:** 10.1155/2014/831073

**Published:** 2014-02-09

**Authors:** Weimin Wang, Yongle Wu, Yuanan Liu

**Affiliations:** ^1^School of Electronic Engineering, Beijing University of Posts and Telecommunications, P.O. Box 282, Beijing 100876, China; ^2^Beijing Key Laboratory of Work Safety Intelligent Monitoring, Beijing University of Posts and Telecommunications, Beijing 100876, China; ^3^The State Key Laboratory of Millimeter Waves, Southeast University, Nanjing 210096, China

## Abstract

A novel coupled-line structure is proposed to design dual-band and high-power Gysel power dividers with inherent impedance-transforming functions. Based on traditional even- and odd-mode technique, the analytical design methods in closed-form formula are obtained and the accurate electrical parameters analysis is presented. Due to the usage of coupled-line sections, more design-parameter freedom and a wider frequency-ratio operation range for this kind of dual-band Gysel powder divider are obtained. Several numerical examples are designed and calculated to demonstrate flexible dual-band applications with different impedance-transforming functions. A practical microstrip power divider operating at 2 GHz and 3.2 GHz is designed, fabricated, and measured. The good agreement between the calculated and measured results verifies our proposed circuit structure and analytical design approach.

## 1. Introduction

Power dividers can be used to design high-power parallel power amplifiers and high-gain antenna arrays [1]. In order to satisfy excellent heat sinking requirement in high-power applications, Gysel power dividers [2] are preferred in practical implementations, compared with Wilkinson power dividers [3]. In the past years, with the rapid development of several wireless communication standards, more and more applications require dual- or multi-band microwave components which results in new modified schemes proposed for dual-band power dividers [4–14]. These dual-band power dividers include Wilkinson types [4–10] and Gysel types [11–14]. Recently, other new kinds of Gysel power dividers are proposed in [15–18]; however, these new power dividers in [15–18] cannot satisfy flexible dual-band applications. Although the flexible dual-band design can be obtained from the proposed Gysel power dividers in [11–13], the inherent impedance-transforming functions cannot be achieved easily. Note that the dual-band Gysel power divider in [14] has narrow operating bandwidth. In addition, the coupled-line circuits are not used in [14].

Coupled lines have many advantages, such as compact structure and flexible design parameters, which have been widely used in impedance transformers [19] and couplers [20]. In this paper, a novel dual-band coupled-line Gysel power divider *with inherent impedance-transforming functions* is proposed. Because of using coupled-line sections, additional design parameters make the proposed structure have more design freedom and the corresponding circuit becomes more simple and compact. Based on the traditional even- and odd-mode analysis, rigorous closed-form design equations and available constraint condition are derived. Different from the previous coupled-line Gysel power divider in [13], this paper gives exact closed-form analytical design equations and inherent impedance-transforming functions. This novel Gysel power divider which has arbitrary different terminated impedances of the source and the load ports presents a wider frequency-ratio range compared to [11, 12]. The available design parameters of typical examples are presented for convenient applications. Finally, numerical examples are presented to demonstrate the flexible dual-band applications and impedance-transforming functions. A practical microstrip power divider operating at both 2 GHz and 3.2 GHz is designed, fabricated, and measured. The calculated and measured results verify our proposed idea.

## 2. The Proposed Circuit and Analytical Design Approach

**Figure 1:**
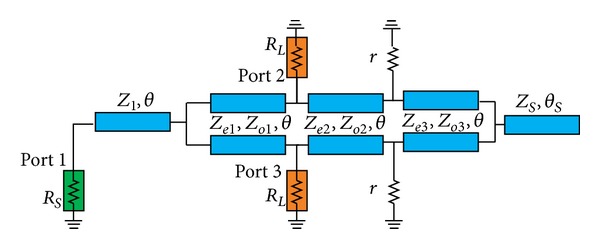
The circuit configuration of the proposed impedance-transforming dual-band coupled-line Gysel power divider.

The circuit configuration of the proposed impedance-transforming dual-band coupled-line Gysel power divider is shown in Figure 1. The structure consists of three sections of coupled lines, an extension line *Z*_1_ at the input port, a single open-circuit stub line *Z*_*S*_ at the right side, and two directly grounded isolation resistors *r*. These coupled lines with characteristic impedances *Z*_*ei*_ and *Z*_*oi*_, where *i* = 1,2, 3, make the structure simplified and compact. All the coupled lines have the same electrical length *θ*.

**Figure 2:**
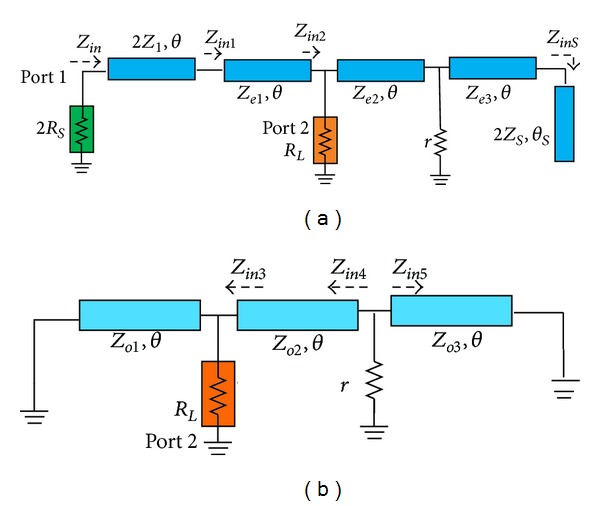
Simplified equivalent circuit of the proposed power divider under (a) even- and (b) odd-mode excitations.

Based on the conventional even- and odd-mode analysis [1], the complicated circuit in Figure 1 can be simplified to the equivalent half circuits in Figure 2, where the even- and odd-mode characteristic impedances of coupled lines are effectively separated for simplicity. Since the divider should be matched at all ports and should divide the input power equally into the two output ports without any power loss at two different frequencies, the even-mode half circuit of the divider should be matched at both ports with lossless transmission. As a result, the isolation resistor *r* does not have any currents and voltages under even-mode excitation. That is to say, the isolation resistor *r* in Figure 2(a) should be bypassed to ground. Hence, the following relationship of the parameters in Figure 2(a) must be satisfied:

(1) ZinS=2ZSjtanθ,Ze3ZinS+jZe3tanθZe3+jZinStanθ=0,

(2) Zin2=jZe2tanθ||RL=jZe2tanθRLjZe2tanθ+RL,Zin1=Ze1Zin2+jZe1tanθZe1+jZin2tanθ,Zin=2Z1Zin1+j2Z1tanθ2Z1+jZin1tanθ=2RS.

Similarly, with the odd-mode excitation, the circuit of the divider shown in Figure 2(b) should be satisfied with the ideal matching and isolation performance at the output ports 2 and 3 shown in Figure 1. Therefore, the reflection coefficients in Figure 2(b) are equal to zero. The relationship of the parameters include odd-mode characteristic impedances in Figure 2(b) that can be derived as

(3) Zin3=jZo1tanθ||RL=jZo1tanθRLjZo1tanθ+RL,Zin4=Zo2Zin3+jZo2tanθZo2+jZin3tanθ,r=Zin4||Zin5=Zin4||jZo3tanθ=jZo3tanθZin4Zin4+jZo3tanθ.

Assuming that the center frequencies *f*_1_ and *f*_2_ = *pf*_1_, where *p* ≥ 1, the simplest conditions [9, 13] for dual-band applications should be described as

(4) θf1=π1+p, (for  f1),θf2=pπ1+p, (for  f2).

Assuming *θ* = *θ*_*f*1_ is known, there are 11 design variables with 8 rigorous equations in (1), (2), and (3), namely, *Z*_1_, *Z*_*e*1_, *Z*_*o*1_, *Z*_*e*2_, *Z*_*o*2_, *Z*_*e*3_, *Z*_*o*3_, *Z*_*S*_, *R*_*L*_, *R*_*S*_, and *r*. From (1), we can obtain

(5) Ze3=2ZStan2θ.

In (5), there is one degree of freedom in the choice of *Z*_*S*_ and *Z*_*e*3_. From (2) and (3), we can obtain that there are five degrees of freedom in the choice of *Z*_1_, *Z*_*e*1_, *Z*_*o*1_, *Z*_*e*2_, *Z*_*o*2_, *Z*_*o*3_, *R*_*L*_, *R*_*S*_, and *r* which offers more flexibility in the design and fabrication of the proposed Gysel power divider than the traditional ones. Assuming *Z*_*e*1_, *Z*_*o*1_, *R*_*L*_, *R*_*S*_, and *r* are known, from (2), we can obtain even-mode characteristic impedances described as

(6) C1Z14+C2Z13+C3Z12+C4Z1+C5=0,Ze2=(2Z1Ze1RLRS−Ze12RLRStan2θ)×(−2Z12Ze1tan2θ+Z1(2RLRS−Ze12)tan2θ+Ze1RLRStan2θ)−1,

where

(7) C1=16Ze1RLtanθA3,C2=8Ze1RLtanθA4−8RLtanθ3A1+8Ze12RLtanθA3,C3=4Ze1RLtanθA5−4RLtanθ3A2  +4Ze1RLtanθ(RL−2RS)A1+4Ze12RLtanθA4,C4=2Ze12RLtanθA5+4Ze12RStanθ3A1  +2Ze1tanθ(RL−2RS)A2,C5=2Ze12RStanθ3A2,A1=2Ze1RLRS,A2=−2Ze12RLRStan2θ,A3=−Ze1tan2θ,A4=2RLRStan2θ−Ze12tan2θ,A5=2Ze1RLRStan2θ.

According to the solution of a quartic equation in [21], we can obtain the following solutions:

(8) Z1(1)=C24C1−12Δ1−12(Δ2−4C1C2C3−C23−8C12C44C13Δ1),Z1(2)=C24C1−12Δ1+12(Δ2−4C1C2C3−C23−8C12C44C13Δ1),Z1(3)=C24C1+12Δ1−12(Δ2+4C1C2C3−C23−8C12C44C13Δ1),Z1(4)=C24C1+12Δ1+12(Δ2+4C1C2C3−C23−8C12C44C13Δ1),Ze2=(2Z1Ze1RLRS−Ze12RLRStan2θ)×(−2Z12Ze1tan2θ+Z1(2RLRS−Ze12)tan2θ+Ze1RLRStan2θ)−1,

where

(9) Δ1=C224C12−2C33C1+Δ3+Δ4,Δ2=C222C12−4C33C1−Δ3−Δ4,Δ3=Δ59Δ4C12,Δ4=23Δ53C1Δ6+−4(Δ5)3+Δ623,Δ5=C32−3C2C4+12C1C5,Δ6=2C33−9C2C3C4+27C1C42+27C22C5−72C1C3C5.

From (3), odd-mode characteristic impedances can be obtained:

(10) Zo2=(−Zo1RL2+Zo1  ×RL4−RL(Zo12tan2 θ+RL2)(RL−r sec2 θ))  ×(Zo12tan2 θ+RL2)−1,Zo3=rZo1Zo2Zo1RL−Zo1r+Zo2RL.

From (5), (7), (8), (9), and (10), once the parameters *Z*_*S*_, *Z*_*e*1_, *Z*_*o*1_, *R*_*L*_, *R*_*S*_, *θ*, and *r* are determined, other parameters such as *Z*_*e*2_, *Z*_*o*2_, *Z*_*e*3_, *Z*_*o*3_, and *Z*_1_ can be easily be synthesized. In consequence, the design method of this proposed coupled-line Gysel power divider for dual-band applications is analytical. Since the values of *R*_*L*_ and *R*_*S*_ are determined by special practical requirements, this presented design approach can satisfy inherent source-to-load impedance-transforming functions.

## 3. Parameters Analysis and Circuit Simulation

**Figure 3:**
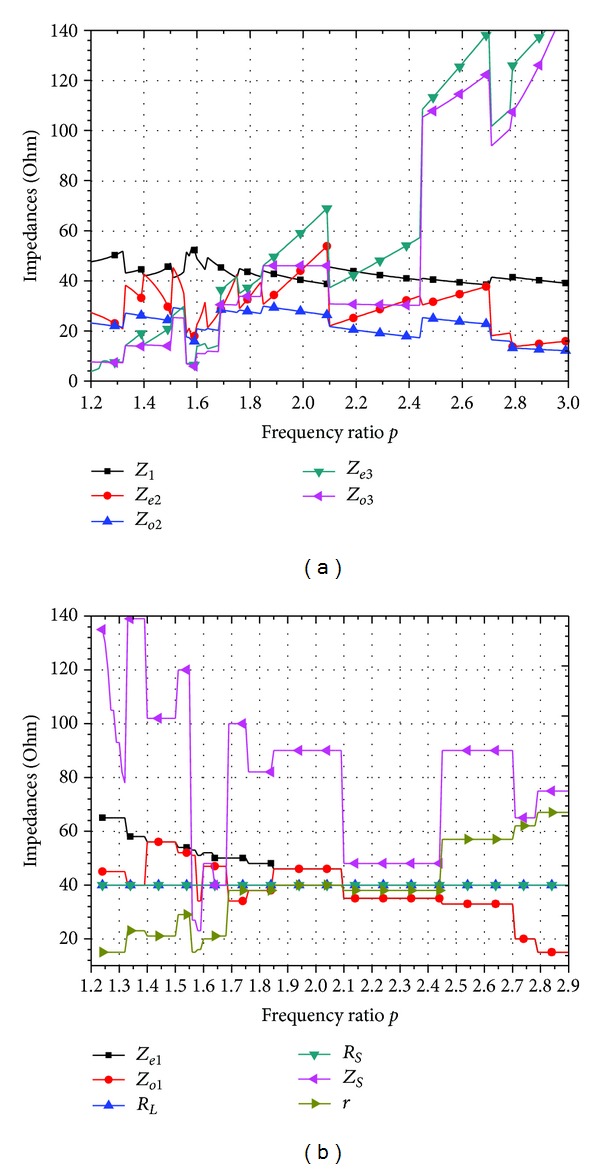
Typical design parameters of the proposed dual-band power divider with *R*_*L*_ = *R*_*S*_ = 40 *Ω*. (a) Values of 5 variables: *Z*_1_, *Z*_*e*2_, *Z*_*o*2_, *Z*_*e*3_, and *Z*_*o*3_. (b) Values of 6 free variables: *Z*_*e*1_, *Z*_*o*1_, *R*_*L*_, *R*_*S*_, *Z*_*S*_, and *r*.

**Figure 4:**
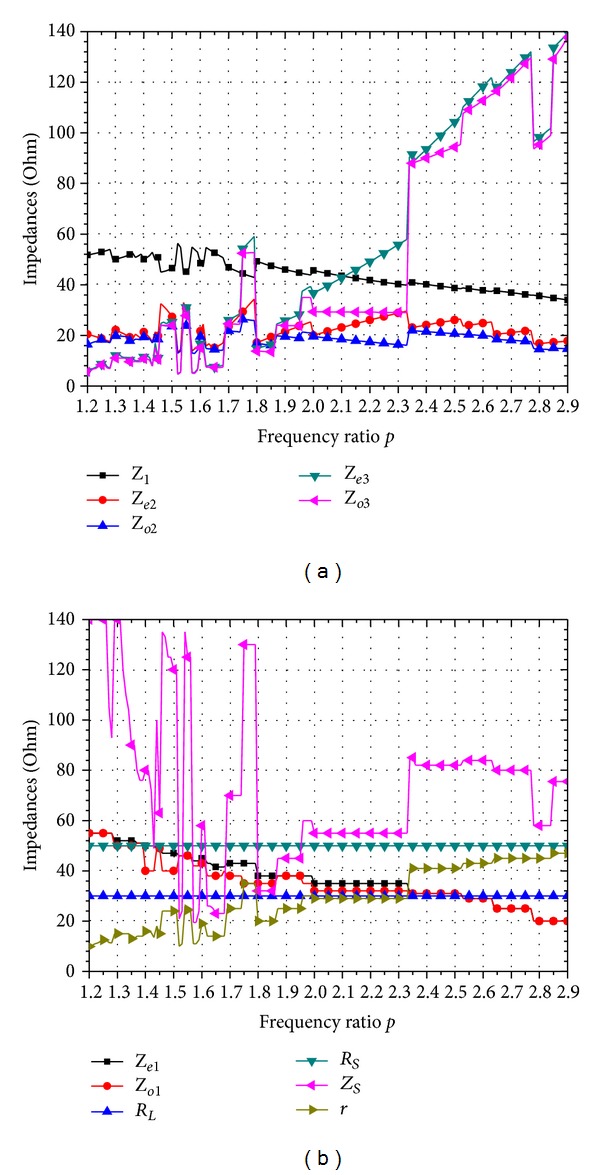
Typical design parameters of the proposed dual-band power divider with *R*_*S*_ = 50 *Ω*, *R*_*L*_ = 30 *Ω*. (a) Values of 5 variables: *Z*_1_, *Z*_*e*2_, *Z*_*o*2_, *Z*_*e*3_, and *Z*_*o*3_. (b) Values of 6 free variables: *Z*_*e*1_, *Z*_*o*1_, *R*_*L*_, *R*_*S*_, *Z*_*S*_, and *r*.

**Figure 5:**
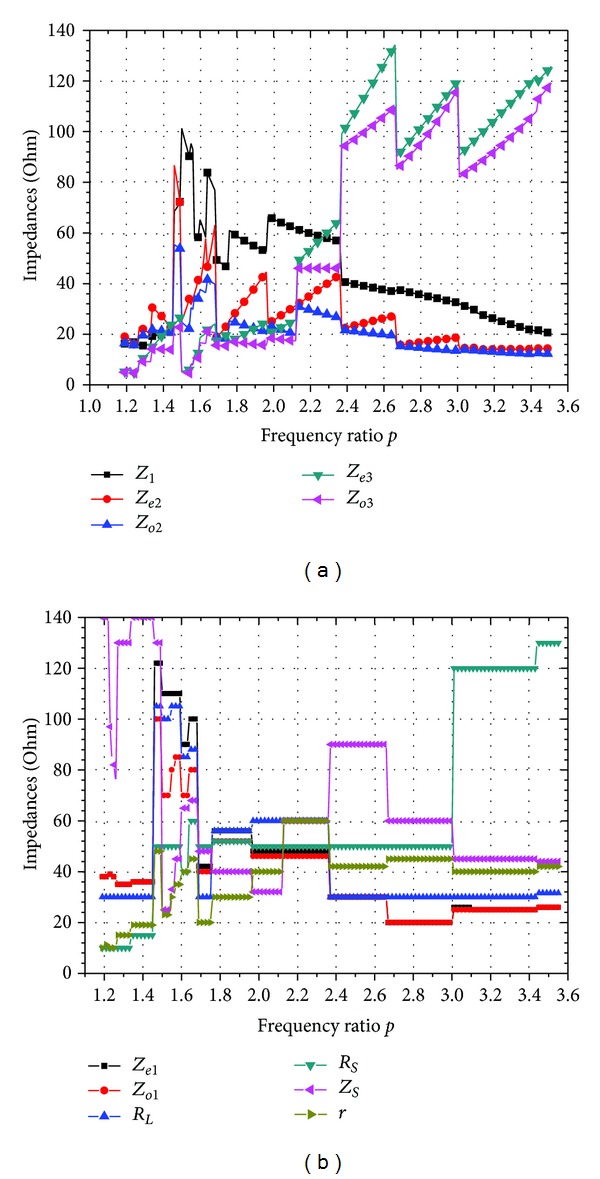
Typical design parameters of the proposed dual-band power divider with arbitrary *R*_*S*_ and *R*_*L*_ values. (a) Values of 5 variables: *Z*_1_, *Z*_*e*2_, *Z*_*o*2_, *Z*_*e*3_, and *Z*_*o*3_. (b) Values of 6 free variables: *Z*_*e*1_, *Z*_*o*1_, *R*_*L*_, *R*_*S*_, *Z*_*S*_, and *r*.

Since there is more degree of freedom of the variables, the design parameters of the proposed divider are calculated under various operating conditions using the design equations of the previous section. Figures 3, 4, and 5 show the design parameters varying with the frequency ratio when the source impedance *R*_*S*_ at port 1 and the load impedance *R*_*L*_ at the output ports 2 and 3 get different values.

There are three conditions of the values of *R*_*S*_ and *R*_*L*_. One condition is *R*_*S*_ is equal to *R*_*L*_ (shown in Figure 3) which represents most of the practical applications. Another condition is that *R*_*S*_ is unequal to *R*_*L*_ but they still get fixed values (shown in Figure 4). The last one is that the values of *R*_*S*_ and *R*_*L*_ vary with the frequency ratio (shown in Figure 5). Figures 3 and 4 show the calculation results when *R*_*S*_ = *R*_*L*_ = 40 *Ω* and *R*_*S*_ = 50 *Ω*, *R*_*L*_ = 30 *Ω*, respectively. From Figures 3 and 4, we can see that the frequency ratio of the proposed divider is 1.2 < *p* < 2.9 when *R*_*S*_ = *R*_*L*_ = 40 *Ω*, and the frequency ratio is 1.24 < *p* < 2.9 when *R*_*S*_ = 50 *Ω*, and *R*_*L*_ = 30 *Ω*. From these figures, we can obtain that the proposed method provides a wider frequency ratio than that (1.35 < *p* < 2.7) reported in [11, 12]. *R*_*S*_ and *R*_*L*_ may get various kinds of values in practical applications according to special requirements. If the parameters including *R*_*S*_ and *R*_*L*_ get suitable values, the proposed method is applicable to the dual-band operation over a very wide frequency-ratio range compared with the situations shown in Figures 3 and 4. Figure 5 shows that when the values of *R*_*S*_ and *R*_*L*_ vary from 10 *Ω* to 130 *Ω*; the frequency ratio can reach from 1.19 to 3.51 which is significantly wider than that mentioned above. It is easy to see that with different value combinations of *R*_*S*_ and *R*_*L*_, different curves as shown in Figure 5 can be obtained. This makes the presented Gysel divider much more flexible in the design and fabrication by providing a variety of alternative solutions for the parameters in fixed dual-band operation.

In fact, the proposed coupled-line Gysel power divider theoretically includes the dividers given in [11, 12]. When *Z*_*e*1_ = *Z*_*o*1_, *Z*_*e*2_ = *Z*_*o*2_, *Z*_*e*3_ = *Z*_*o*3_, and *R*_*S*_ = *R*_*L*_, this proposed divider turns into the one in [11]. When *Z*_1_ = 50 *Ω* and *R*_*S*_ = *R*_*L*_ = 50 *Ω*, this proposed divider represents the one in [12]. Therefore, this proposed Gysel power divider is a generalized power divider with increased design freedom and a wider frequency-ratio range.

**Figure 6:**
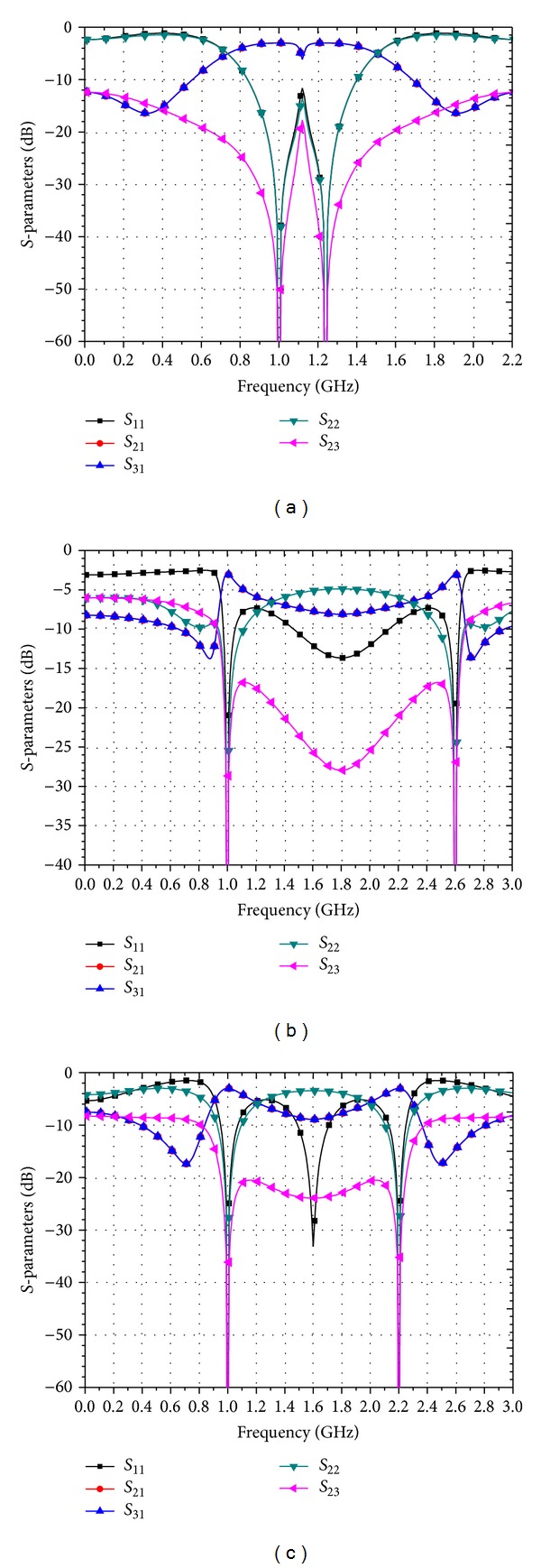
Ideal scattering parameters of three typical dual-band coupled-line power dividers with: (a) *R*_*S*_ = *R*_*L*_ = 40 *Ω* and *p* = 1.24; (b) *R*_*S*_ = 50 *Ω*, *R*_*L*_ = 30 *Ω* and *p* = 2.6; and (c) *R*_*S*_ = 50 *Ω*, *R*_*L*_ = 60 *Ω*, and *p* = 2.2.

Figure 6 presents three groups of numerical examples when *R*_*S*_ = *R*_*L*_ = 40 *Ω*, and *p* = 1.24 (Figure 6(a)); *R*_*S*_ = 50 *Ω*, *R*_*L*_ = 30 *Ω*, and *p* = 2.6 (Figure 6(b)); and *R*_*S*_ = 50 *Ω*, *R*_*L*_ = 60 *Ω*, and *p* = 2.2 (Figure 6(c)), respectively. The simulation results show that the performance of the proposed Gysel power divider operating at two different frequencies is perfect although the terminated impedances *R*_*S*_ and *R*_*L*_ are different and the frequency-ratio *p* is flexible.

## 4. Microstrip Experiment and Measurement

To verify the circuit structure and analytical design approach of our proposed dual-band Gysel power divider, a prototype microstrip dual-band power divider has been designed and fabricated. It is designed for the dual-band operation with a frequency ratio of 1.6 at *f*_1_ = 2 GHz and *f*_2_ = 3.2 GHz. The terminated source impedance *R*_*S*_ at port 1 and the load impedance *R*_*L*_ at the output ports 2 and 3 are randomly chosen as *R*_*S*_ = 30 *Ω* and *R*_*L*_ = 50 *Ω*. In order to match the source impedance *R*_*S*_ = 30 *Ω* to the standard impedance *R*_*O*_ = 50 *Ω*, a small dual-frequency transformer in two section transmission line given in [22] is chosen. Assuming the characteristic impedances of the two section transmission line are *Z*_1_′ and *Z*_2_′, we can obtain

(11) Z1′=RS2 tan2 θ(RO−RS)+[RS2 tan2 θ(RO−RS)]2+RS3RO,Z2′=RSROZ1′,

where

(12) θ=180°1+p=180°1+1.6=69.2308°,RO=50 Ω.

From (11) and (12), *Z*_1_′ = 34.7253 *Ω* and *Z*_2_′ = 43.1962 *Ω* can be calculated.

**Figure 7:**
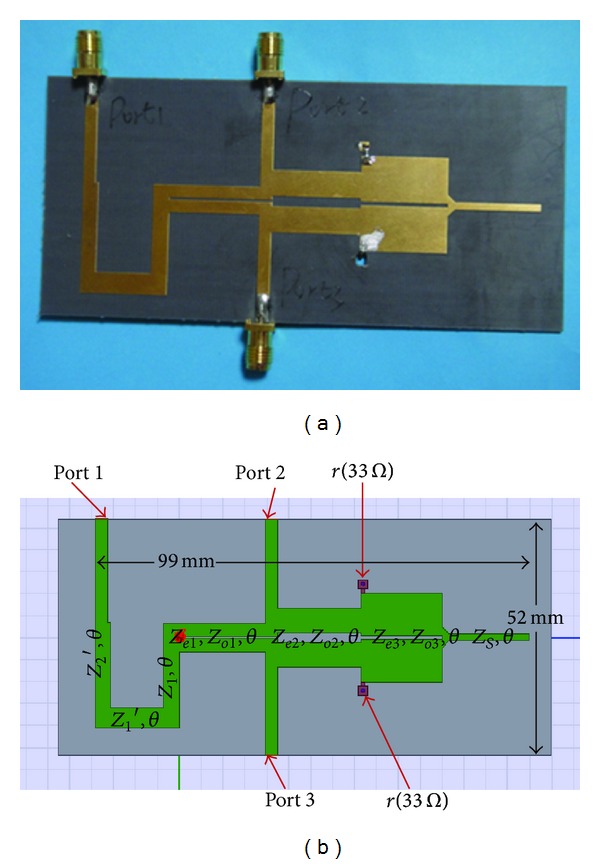
Fabricated coupled-line Gysel power divider operating at both 2 and 3.2 GHz. (a) Photograph and (b) 3D model.

**Figure 8:**
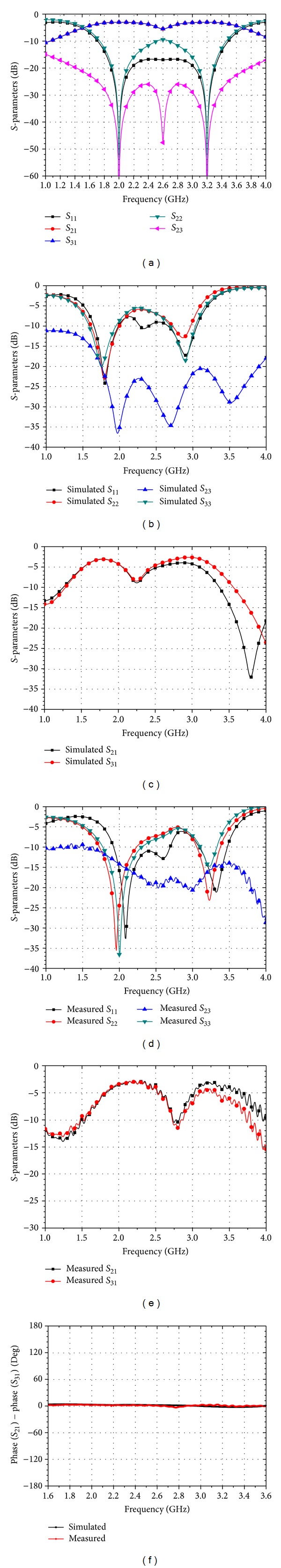
Scattering parameters of the fabricated divider shown in Figure 7: (a) calculated results, (b) and (c) full-wave simulated results, (d) and (e) measured results, and (f) simulated and measured phase differences results.

F4B with a dielectric constant of 2.65 and a thickness of 1 mm is used as the substrate. The coupled lines are implemented by using microstrip technology. All the lines are designed with the same electrical length of *θ* = 69.2308° at *f*_1_ = 2 GHz. The impedances of the transmission and coupled lines are calculated as *Z*_1_ = 41.5654 *Ω*, *Z*_*e*1_ = 57.5 *Ω*, *Z*_*o*1_ = 40 *Ω*, *Z*_*e*2_ = 31.3839 *Ω*, *Z*_*o*2_ = 28.7344 *Ω*, *Z*_*e*3_ = 20.1363 *Ω*, *Z*_*o*3_ = 17.9188 *Ω*, and *Z*_*S*_ = 70 *Ω*, *r* = 33 *Ω*.  Figure 7(a) shows a photograph of the fabricated power divider. Full-wave simulation is made for the power divider. The 3D HFSS model and the size of the divider is shown in Figure 7(b). The calculated, full-wave simulated and measured results are shown in Figure 8. From *S*_11_ curve in Figures 8(b) and 8(d), we can obtain that the two center frequencies of the simulated power divider are 1.8 GHz and 2.9 GHz, and the measured results are 2.09 GHz and 3.32 GHz, respectively, which are both very close to the theoretical values of 2 GHz and 3.2 GHz. The simulated port isolation values of *S*_23_ are all below −15 dB in the available band from 1.6 GHz to the whole frequency band (including 1.8 GHz to 2.9 GHz). The measured magnitude values of *S*_23_ are nearly all below −10 dB in the whole frequency band. These influences are acceptable in practical application. The simulated and measured results verify the availability of the proposed structure and the corresponding analytical design approach.

## 5. Conclusions

A novel impedance-transforming coupled-line Gysel power divider is proposed for dual-band and high-power applications in this paper. The closed-form formulas to determine its design parameters have been given. The use of coupled lines in this new divider provides the advantages of a simplified structure, compact size, a wider frequency-ratio range, and additional design freedom. Increased design freedom causes a variety of alternative solutions for the parameters which makes the design and fabrication more flexible. The circuit calculation, full-wave simulation, microstrip fabrication, and measurement of a prototype Gysel power divider verify the proposed structure and its related design theory. It can be believed that this kind of novel impedance-transforming coupled-line dual-band Gysel power divider can be widely used in various high-power power amplifiers and high-performance antenna arrays, especially for dual-band circuits and systems.
